# Estimates of air pollution in Delhi from the burning of firecrackers during the festival of Diwali

**DOI:** 10.1371/journal.pone.0200371

**Published:** 2018-08-13

**Authors:** Dhananjay Ghei, Renuka Sane

**Affiliations:** 1 Department of Economics, University of Minnesota, Minneapolis, Minnesota, United States of America; 2 National Institute of Public Finance and Policy, Delhi, India; University of Maryland at College Park, UNITED STATES

## Abstract

Delhi has had the distinction of being one of the most polluted cities in the world, especially in the winter months from October—January. These months coincide with the religious festival of Diwali. It is argued that air quality gets worse in the aftermath of Diwali on account of firecrackers that get burned during the festival. We use hourly data on PM 2.5 particulate concentration from 2013 to 2017 to estimate the Diwali effect on air quality in Delhi. We improve on existing work by using the event study technique as well as a difference-in-difference regression framework to estimate the Diwali effect on air quality. The results suggest that Diwali leads to a small, but statistically significant increase in air pollution. The effect is different across locations within Delhi. To our knowledge, this is the first causal estimate of the contribution of Diwali firecracker burning to air pollution.

## Introduction

In 2014, Delhi became the most polluted city in the world [1, 2]. Since then it has continued to be in the list of the world’s most polluted cities [3]. Air pollution is worse in the winter months (October—January) as particles remain suspended in the air for longer duration of time due to the lower temperature, wind speed as well as higher relative humidity. In early November, farmers in the neighbouring states of Punjab and Haryana burn the stubble from the previous harvest to prepare land for the next sowing season, and the smoke is carried to Delhi contributing to the smog [4].

These winter months coincide with a very important religious festival in India, namely, Diwali. It is argued that air quality gets worse in the aftermath of Diwali, on account of firecrackers that get burned during the festival. The link between firecracker burning and air pollution has been established in other regions (for example, [5]). This has resulted in calls for banning the sale of firecrackers, and in 2017, the Supreme Court of India did order such a ban.

The question of how much does air pollution increase because of firecracker burning is an important one, because measures such as the ban on the sale of firecrackers impose significant costs in the form of reduced livelihoods of people in the trade.

Existing research on the impact of Diwali on air quality in India has focused on measuring the concentration of pollutants in the air around Diwali [6], [7], [8], [9], [10], [11], [12], [13], [14]. For example, [8] found Diwali day 24 hour average concentrations, in Lucknow, to be 2.49 and 5.67 times higher when compared with the concentration of pre-Diwali and normal day respectively. In addition, they found *SO*_2_ concentrations to be 1.95 and 6.59 times higher compared to the concentration of pre-Diwali and normal days. [7] investigated metal concentrations and found that significant amounts of metals released in air contributed to heavy air pollution on Diwali. More recently [15] analyse PM 10 loads and chemical compounds a few days prior, during and post Diwali and find that firework emissions significantly affect air quality.

However, it is possible that the bad air that we see in Delhi at the time of Diwali is just the bad air quality in winter, and is not causally impacted upon by Diwali. The studies mentioned above make progress on measurement, and show correlations between firecrackers and Diwali, but do not conclusively establish the *causal* relation between them. The studies also make measurements at local weather stations, but are not able to evaluate the impact on multiple stations within a city at the same time.

In this paper, we use hourly data from 2013 to 2017 to estimate the Diwali effect on air quality, in particular PM 2.5 particulate concentration, in Delhi. We improve on existing work by using the event study technique as well as a difference-in-difference regression framework to estimate the “Diwali” effect on air quality. We find that Diwali leads to a small, but statistically significant increase in air pollution. The effect is different across locations within Delhi. To our knowledge, this is the first *causal* estimate of the contribution of Diwali firecracker burning to air pollution.

The health implications of poor air quality [16] [17] are leading to pressure on the government in Delhi to respond to this crisis. There is a clamour for regulatory interventions that will yield clean air. In January 2016, the Delhi government constituted a policy to restrict cars on roads on certain days (known as the odd-even rule). In the same year, post Diwali, the government declared a public health emergency and shut down schools as well as power plants around Delhi temporarily. In 2017, nine days before Diwali, the Supreme Court of India banned the sale of fireworks in Delhi.

These interventions are often arbitrary and knee-jerk responses to an impending crisis. As a consequence, they have little effects. For example, [18] show that the 2016 odd—even rule for vehicles was not effective in reducing measurable PM 2.5 pollution in Delhi. Only when we are able to marshal evidence in a systematic way about the extent and nature of the problem, will we be able to design and deliver a response. Estimation is also important as it helps policy makers arrive at a cost-benefit analysis of particular intervention.

The measurement of air pollution in Delhi has begun on a small scale. Granular and high frequency data was made available following 2013 when standardised monitors were placed in different parts of the city to measure pollution levels. Our paper uses the relatively recently available data to contribute to knowledge on air pollution.

## Methods

### Ethics statement

All the meteorological data collected at the five monitoring sites used in this study are publicly available on the internet, and no specific permissions are required to access these sites.

### The use of PM 2.5

There are many pollutants in the air such as carbon monoxide (CO), nitric oxide (NO), nitrogen dioxide (NO2), ozone (O3). The worst among these is small particulate matter, or PM 2.5, a mixture of solid and liquid droplets floating in the air whose diameter is less than 2.5 micrometers. PM 2.5 particles are produced from all types of combustion, including motor vehicles and power plants and some industrial processes.

The health impact from pollution is a complex transform of exposure to all pollutants. However, of the pollutants, PM 2.5 particles are considered the most harmful as they are able to enter deep into the respiratory tract, reaching the lungs. This can cause short-term health effects such as eye, nose, throat and lung irritation, coughing, sneezing, runny nose and shortness of breath, and in the long-term can affect lung function and worsen medical conditions such as asthma and heart disease. We, therefore, narrow our attention to the measure of PM 2.5. The unit of measurement of PM 2.5 is *μ*g/*m*^3^.

### Data sources

We fetch raw PM 2.5 values from two data sources on pollution in Delhi. The first is data from the US Embassy based in Chanakyapuri. The second is from the Central Pollution Control Board (CPCB) that puts out data for various locations across India.

While CPCB has several monitors in Delhi, we selected the four locations that provided us with the most consistent dataset. We thus have data for five locations: 1) R. K. Puram in South West Delhi which is a residential area, 2) Punjabi Bagh in West Delhi, also a residential area 3) Mandir Marg in Central Delhi, 4) US Embassy in the diplomatic enclave in Central Delhi, and 5) Anand Vihar in East Delhi, which is adjacent to an industrial area.

In addition to PM 2.5, we also extract hourly data on wind, temperature and relative humidity for all the locations on the CPCB website. This is in the form of several dropboxes where one has to select the name of the city, and station, the desired time-period as well as the indicators for which data is required. The data on the additional variables is not available for the Chanakyapuri location.

We use hourly data from the locations mentioned above for a time period from January 2013 to May 2017. It should be noted that values are missing from certain sections of the data. These missing observations are excluded from our analysis. We winsorise 1% tail of the observations to remove extreme values.

**Fig 1:**
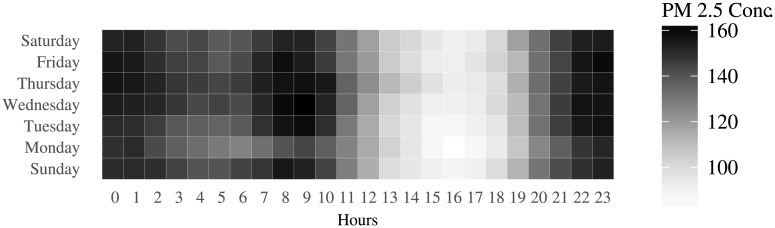
Week effect in pollution levels.

Fig 1 shows the variation in hourly pollution levels during different days of a week. Darker colors represent increased PM 2.5 matter in the air. Regardless of the day, pollution levels are low during the day, but start increasing post 18:00 hours. and remain elevated till 09:00 hours of the next day. The average PM 2.5 concentration across all days from 18:00 to 09:00 the next day is 140 *μ*g/*m*^3^, whereas the average PM 2.5 concentration from 09:00 to 18:00 is 108 *μ*g/*m*^3^. PM 2.5 levels in the range of 101-200 can cause breathing discomfort to anyone with prolonged exposure to the air during these times.

**Fig 2:**
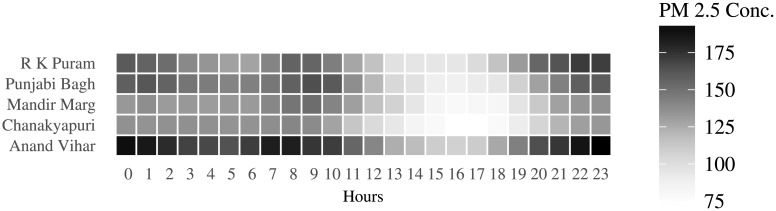
Month effect in pollution levels.

Fig 2 shows the hourly variation in pollution levels during different months of the year. Note that the scale for this figure is different from that used in Fig 1. The monsoon months of July—September have the lowest levels of PM 2.5 particulate concentration. Larger particles are settled in few hours due to gravity, but smaller particles such as PM 2.5 are removed by precipitation. Winters have the highest levels of PM 2.5 matter in the air, on account of low wind speed and high relative humidity.

**Fig 3:**
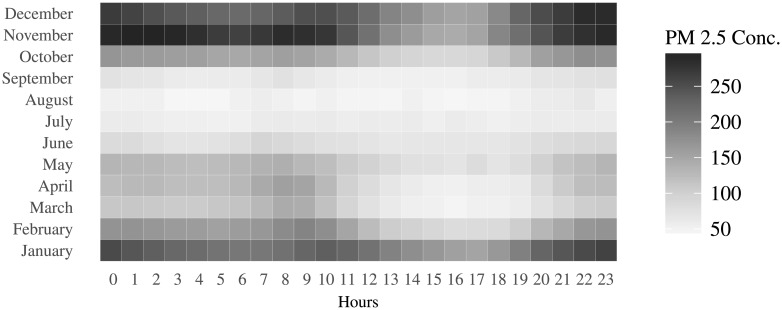
Location effect in pollution levels.

Fig 3 shows the hourly variation in pollution levels across the five locations. The diplomatic enclave of Chanakyapuri seems to perform better than other areas of Delhi. Anand Vihar in East Delhi has the highest pollution levels amongst the 5 different locations, and has severe levels of air pollution in the night. There is a strong location effect on pollution levels. This can be attributed to varying population densities of these locations as well as the proximity to industries.

### The festival of Diwali

Diwali is an important Hindu religious festival celebrated over a four-five day period. The main day of Diwali is called “Lakshmi Puja”. This is celebrated by the burning of firecrackers, which typically begins around 18:00 hours.

Diwali does not fall on the same date every year as it is based on the Hindu lunar calendar. As a result it is a “moving date” across different years. In the period between January 2013 and May 2017, Diwali occurred on the following dates: Nov 4, 2013, Oct 22, 2014, Nov 11, 2015, Oct 30, 2016.

### Estimation

We estimate the Diwali effect using two methods. The first of these is the event study methodology. This methodology is generally used in the field of finance to measure the impact of a specific event on the value of the firm [19]. We adopt the same methodology to evaluate the impact of Diwali on PM 2.5 particulate concentration.

The day of the Lakshmi Puja is taken as the event day. We have 4 Diwali events and 4 locations. This gives us a total of 16 events. The Chanakyapuri location is dropped as climate data about this location is not available.

We take the mean of the hourly pollution levels on each date as a proxy for daily series. Next, we calculate the percentage change in PM 2.5 concentration levels by differencing the logarithm of PM 2.5 values. These are then re-indexed to show the cumulative change over a 10 day window.

Next, we perform an hourly event study using the same methodology. Here, we use 1800 hours on the day of Diwali as *t* = 0 event time. Once again, we have a total of 16 events.

As discussed earlier, it is possible that the bad air that we see in Delhi at the time of Diwali is just the bad air quality in winter, and is not causally impacted upon by Diwali. In early November, farmers in the neighbouring states of Punjab and Haryana burn the stubble from the previous harvest to prepare land for the next season, and the smoke is carried to Delhi contributing to the smog [4].

The opportunity to identify a Diwali effect comes from the fact that Diwali is a ‘moving holiday’ which takes place on a different day of each year. If this were not the case, it would be strongly correlated with changing climate, or with stubble burning. We estimate the Diwali effect across locations using a difference-in-difference regression. The model is as follows:

$$\begin{array}{c} PM_{iht}=\beta_{0}+\beta_{1}L_{i}+\beta_{2}D_{t}+\beta_{3}D_{t}L_{i}+\beta_{4}WS_{iht}+\beta_{5}RH_{iht}+\beta_{6}AT_{iht}+\beta_{7}m_{t}+\gamma_{h}+\epsilon_{iht} \end{array}$$ (1)

where, *i* is location, *h* is hour, *t* is date, *PM*_*iht*_ is PM 2.5 parameter recorded at location *i*, hour *h* and date *t*. *L*_*ht*_ is the dummy for location. *D*_*t*_ is the dummy for Diwali which is 1 on the date of Diwali and 0 otherwise. The Diwali dates constitute the treatment group, while the remaining dates are the control group. *WS*_*iht*_ is wind speed at location *i*, hour *h* and date *t*, *RH*_*iht*_ is relative humidity at location *i*, hour *h* and date *t*, *AT*_*iht*_ is ambient temperature at location *i*, hour *h* and date *t*. Hour (*γ*_*h*_), month (*m*_*t*_) are fixed effects. The regression is restricted to the months of October and November. This ensures that there is no great difference between the ambient air pollution between the Diwali and non-Diwali days. Robust standard errors are used for our analysis throughout.

## Results

### Event study

**Fig 4:**
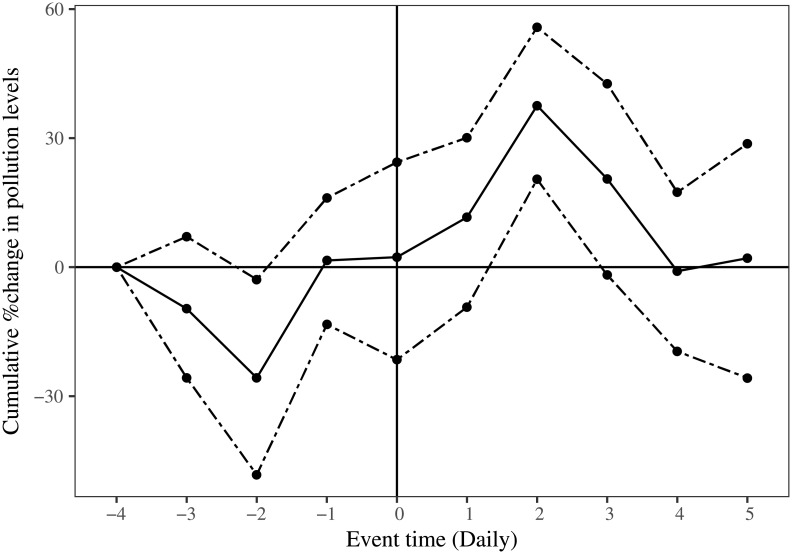
Event study on daily data.

Fig 4 shows the event study on daily data. The solid line represents the average cumulative percentage change in PM 2.5 values during the event window. The dashed line represents the confidence intervals calculated using bootstrapped standard errors. We see that pollution levels start increasing two days before Diwali, and increase till two days after Diwali.

**Fig 5:**
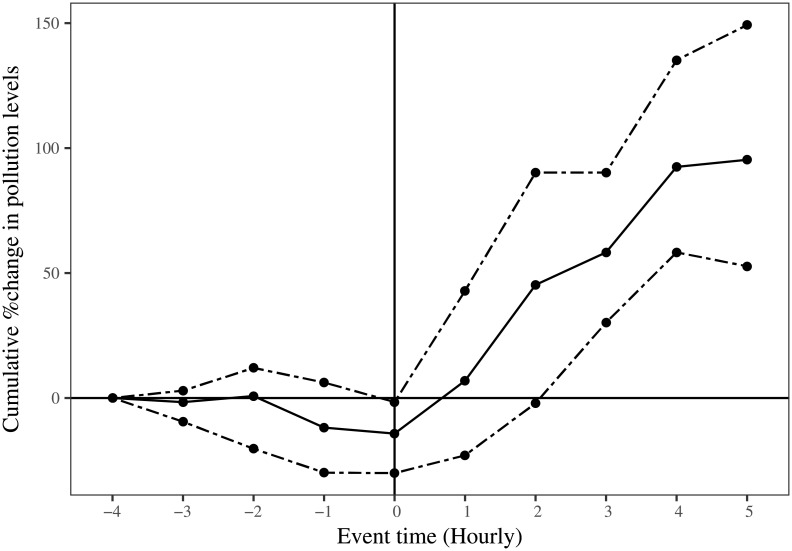
Event study on hourly data.

Fig 5 shows the event study on hourly data. The event time (*t* = 0) is 1800 hours on the day of Diwali across four Diwali events and four locations. As earlier, the solid line represents the average cumulative percentage change in PM 2.5 values during the event window and the dashed lines represent the 95% confidence intervals calculated using bootstrapped standard errors. We see that the pollution levels do not rise before 1800 hours and post the event time there is a statistically significant increase in pollution levels that rise up to approximately 100% in a short span of 5 hours.

### Regression

Table 1 shows the regression results for four different models. Robust clustered standard errors are used throughout. The first model (Column 1) contains only hour effects along with location fixed effects. The second model (Column 2) contains hour and month fixed effects. The third model (Column 3) contains meteorological factors as well and the fourth model (Column 4) contains only meteorological factors.

**Table 1:** Regression results: Part I.

	*Dependent variable:*			
	PM2.5			
	(1)	(2)	(3)	(4)
Constant	284.700*** (7.782)	339.666*** (10.053)	456.354*** (73.609)	480.629*** (44.382)
Mandir Marg	−72.908*** (0.401)	−78.397*** (0.745)	−122.946*** (15.498)	−116.346*** (5.972)
Punjabi Bagh	−49.750*** (0.338)	−54.528*** (0.629)	−71.013*** (9.637)	−71.613*** (3.808)
R K Puram	−45.536*** (0.353)	−49.442*** (0.591)	−77.720*** (13.466)	−77.287*** (5.112)
Diwali	34.031*** (0.918)	15.692*** (0.765)	54.972*** (13.131)	43.041*** (1.013)
Wind speed			−59.179*** (10.354)	−29.246** (13.167)
Relative Humidity			0.834*** (0.156)	−0.252*** (0.026)
Ambient Temperature			−7.914*** (2.988)	−8.391*** (1.333)
Mandir Marg*Diwali	70.543*** (1.004)	77.504*** (0.701)	31.774*** (9.035)	40.048*** (8.325)
Punjabi Bagh*Diwali	76.472*** (1.104)	72.210*** (1.286)	48.988*** (3.202)	55.636*** (1.388)
R K Puram*Diwali	39.406*** (0.359)	24.237*** (1.215)	−50.348*** (1.637)	−50.888*** (1.403)
Hour FE	Y	Y	Y	N
Month FE	N	Y	Y	N
Clustered SEs	Y	Y	Y	Y
Observations	17,380	17,380	4,340	4,340
R^2^	0.174	0.366	0.466	0.351
Adjusted R^2^	0.173	0.364	0.462	0.350
Residual Std. Error	109.666 (df = 17349)	96.130 (df = 17348)	85.054 (df = 4305)	93.483 (df = 4329)
F Statistic	121.971*** (df = 30; 17349)	322.364*** (df = 31; 17348)	110.551*** (df = 34; 4305)	234.622*** (df = 10; 4329)

Note:

*p<0.1;

**p<0.05;

***p<0.01

The two competing models we have is the second (Column 2) and fourth model (Column 4). While the fourth model only accounts for climate factors, the second model accounts for hour and month effects. Since, climate is correlated with the months, the months capture not only the variation due to climate but also captures variation due to other exogenous factors such as stubble burning in the neighboring states of Punjab and Haryana which is a common trend during late October and early November. Given the fine granularity of the data set, model 2 is more representative as it captures not just meteorological factors but also other exogenous factors.

The coefficient on the constant term is the average pollution in Anand Vihar. This is quite high, and as seen in Column (2) was an average of 340 *μ*g/*m*^3^. The other three locations have lower pollution levels on average relative to Anand Vihar.

The coefficient on the Diwali dummy reflects the Diwali effect at Anand Vihar. It is positive and statistically significant across the four different models. The average particulate concentration is 15.7 *μg*/*m*^3^ higher. This is suggestive of the fact that there is certainly a rise in pollution levels in Delhi during Diwali. While this may seem relatively small, it is useful to remember that this is on a base of already high pollution (>300 *μ*g/*m*^3^).

There is a differential effect on Diwali in other locations relative to Anand Vihar on Diwali. For instance, Diwali adds on an average 61.81 (77.504-15.692) *μ*g/*m*^3^ PM 2.5 particulate concentration in the air at Mandir Marg relative to Anand Vihar.

**Fig 6:**
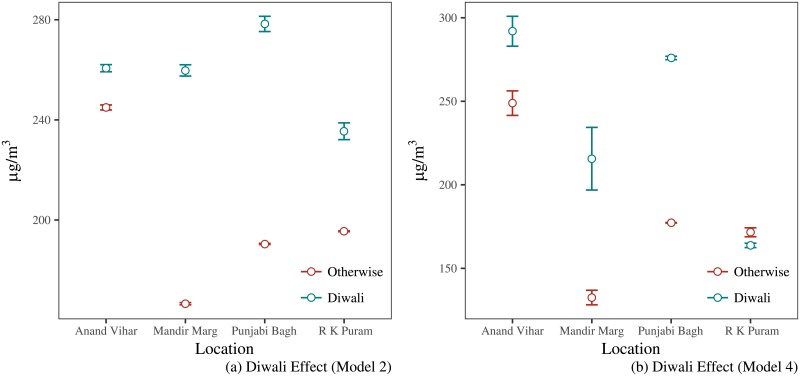
Estimating the Diwali effect: Part I.

Fig 6 shows the estimated marginal effect of Diwali on air pollution levels using Model 2 and Model 4 in Table 1. The effect is the conditional expectation of the PM 2.5 value on different locations during Diwali keeping the other regressors as constant (for categorical variables) and as average (for continuous variables). The figures show that there is an increase in pollution levels during the day of Diwali compared to the control group. The trend is same across all four locations. Tighter confidence intervals suggest that the increase is statistically significant.

It is possible that our results are confounded by fireworks that persist after the Diwali days. We, therefore, also conduct regressions by taking only pre-Diwali days as our control group as a robustness check. In particular, we take 10 days prior to Diwali every year as our control group. We estimate Model 2 and Model 4, and find that the results are as expected and we see that there is an increase in PM 2.5 concentration during Diwali. Results for this regression are available upon request.

One might argue that given the fact that the Diwali effect is sustained for more than 2 days as seen in event study on daily data (Fig 4) this would mean that we are underestimating the impact of Diwali as the control group has on average higher pollution levels since it contains days post Diwali.

We, therefore, divide the Diwali events by months, and use as control groups the same months of the non-Diwali years. Out of the four Diwali events, two of them were in October and two of them were in November. Consider for example, Diwali was in November for 2013 and 2015, thus, we use the control group as November of 2014 and 2016. If the hypothesis is correct, the effect of Diwali should increase since we are now measuring the control group more accurately.

Table 2 shows the regression for October and November. For October, we see an increase in the effect of Diwali when we change the control group. However, this impact is not visible when we consider November.

**Table 2:** Regression results: Part II.

	*Dependent variable:*	
	PM2.5	
	October	November
	(1)	(2)
Constant	209.617*** (6.571)	357.379*** (9.613)
Mandir Marg	−63.321*** (0.182)	−91.233*** (0.958)
Punjabi Bagh	−31.813*** (0.205)	−83.711*** (0.829)
R K Puram	−39.959*** (0.195)	−57.470*** (0.905)
Diwali	100.527*** (1.140)	−24.312*** (1.203)
Mandir Marg*Diwali	37.479*** (1.360)	99.544*** (1.510)
Punjabi Bagh*Diwali	50.248*** (0.940)	108.976*** (1.761)
R K Puram*Diwali	−36.925*** (4.451)	58.822*** (1.412)
Hour FE	Y	Y
Clustered SEs	Y	Y
Observations	4,382	4,335
R^2^	0.152	0.322
Adjusted R^2^	0.146	0.317
Residual Std. Error	93.716 (df = 4351)	97.403 (df = 4304)
F Statistic	26.043*** (df = 30; 4351)	68.125*** (df = 30; 4304)

Note:

*p<0.1;

**p<0.05;

***p<0.01

**Fig 7:**
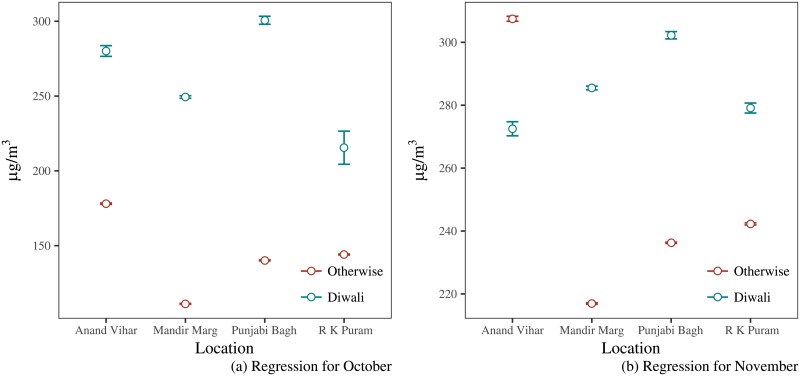
Estimating the Diwali effect: Part II.

Fig 7 shows the estimated effect of Diwali compared to the control group for October and November. The only thing surprising in this case is Anand Vihar where the pollution levels on Diwali are lower compared to the control group. This may be because Anand Vihar is located near the industrial area. Industrial activity comes to a halt before Diwali as workers have holidays which would mean that the pollution from industries is not accounted for and hence, the effect is actually lower compared to the control group.

## Conclusion

Very little is known, at present, about the causal impact of Diwali on air quality. We have begun analysing this question here. Our results suggest that there is a strong location and month effect when examining air quality. Winter months see some of the worst air pollution levels in Delhi. We find that Diwali, on average, leads to increasing pollution levels across all locations in Delhi. Over a period of two days, Diwali adds about 40 *μ*g/*m*^3^ to PM 2.5 particulate concentration. While this number may look small in itself, it is high considering the already poor air quality around the time. There is a wide variation in the effect of firecrackers across locations. It is further important to study the contribution of firecrackers relative to vehicles at the same time. We hope to address this in future research. We also hope that the current study contributes to the cost-benefit analysis of proposed policy measures to reduce air pollution.
