# Correction: Estimates of air pollution in Delhi from the burning of firecrackers during the festival of Diwali

**DOI:** 10.1371/journal.pone.0205131

**Published:** 2018-12-11

**Authors:** Dhananjay Ghei, Renuka Sane

The images for Figs 2 and 3 are incorrectly switched. The image that appears as Fig 2 should be Fig 3, and the image that appears as Fig 3 should be Fig 2. The figure captions appear in the correct order.

**Fig 2:**
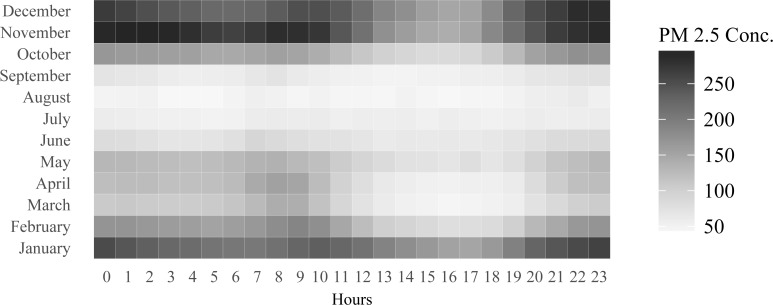
Location effects in pollution levels.

**Fig 3:**
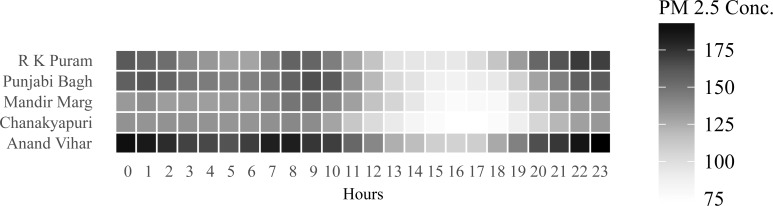
Month effects in pollution levels.
